# Molecular Characterization of HIV-1 Subtype C gp-120 Regions Potentially Involved in Virus Adaptive Mechanisms

**DOI:** 10.1371/journal.pone.0095183

**Published:** 2014-04-30

**Authors:** Alessandra Cenci, Giuseppe D'Avenio, Lara Tavoschi, Michele Chiappi, Simone Becattini, Maria del Pilar Narino, Orietta Picconi, Daniela Bernasconi, Emanuele Fanales-Belasio, Eftyhia Vardas, Hosea Sukati, Alessandra Lo Presti, Massimo Ciccozzi, Paolo Monini, Barbara Ensoli, Mauro Grigioni, Stefano Buttò

**Affiliations:** 1 Istituto Superiore di Sanità, National AIDS Center, Rome, Italy; 2 Istituto Superiore di Sanità, Department of Technology and Health, Rome, Italy; 3 Stellenbosch University, Division of Medical Virology, Stellenbosch, South Africa; 4 National Center Public Health Laboratory, Manzini, Swaziland; 5 Istituto Superiore di Sanità, Department of Infectious, Parasitic and Immunomediated Diseases, Rome, Italy; 6 Lancet Laboratories, Johannesburg, South Africa; 7 University of Biomedical Campus, Rome, Italy; Institute of Infection and Global Health, United Kingdom

## Abstract

The role of variable regions of HIV-1 gp120 in immune escape of HIV has been investigated. However, there is scant information on how conserved gp120 regions contribute to virus escaping. Here we have studied how molecular sequence characteristics of conserved C3, C4 and V3 regions of clade C HIV-1 gp120 that are involved in HIV entry and are target of the immune response, are modulated during the disease course. We found an increase of “*shifting*” putative N-glycosylation sites (PNGSs) in the α2 helix (in C3) and in C4 and an increase of sites under positive selection pressure in the α2 helix during the chronic stage of disease. These sites are close to CD4 and to co-receptor binding sites. We also found a negative correlation between electric charges of C3 and V4 during the late stage of disease counteracted by a positive correlation of electric charges of α2 helix and V5 during the same stage. These data allow us to hypothesize possible mechanisms of virus escape involving constant and variable regions of gp120. In particular, new mutations, including new PNGSs occurring near the CD4 and CCR5 binding sites could potentially affect receptor binding affinity and shield the virus from the immune response.

## Introduction

Human immunodeficiency virus type 1 (HIV-1) gp120 envelope protein is characterized by a remarkable genetic variability [1]–[3], due to extensive replication of the virus and to a high mutation rate of the reverse transcriptase [4]. This variability is largely responsible for the ability of the virus to escape the host immune response, in particular neutralizing activity of antibodies [5]–[8]. The role of the variable regions of gp120 (V1 to V5) in promoting virus escape has been reported by several groups, including ours [9]–[15]. These studies have shown that both amino acid sequence length and number of putative N-glycosylation sites (PNGSs) in the V1, V2 and V4 variable regions of the protein change during the course of the disease, generating HIV variants resistant to neutralizing antibodies. Similar mechanisms of escape have been observed in different HIV-1 subtypes of the M group [10], [15]–[18], although subtype-specific differences may be present [9], [19].

In HIV-1 subtype C, the most widespread HIV clade worldwide and the prevalent one in Southern Africa [20], [21], we and others have shown that during the chronic stage of the disease the V1 and V4 variable regions of gp120 undergo a significant increase of their aminoacid sequence length and new PNGSs are generated [9], [20], while the V5 undergoes changes in its net electrical charge during the course of the disease. These changes may play a role in glycan packing and immune evasion [9], [14], [22].

Few studies have been conducted to date that have investigated the modulation of molecular sequence characteristics of the constant regions of gp120 during the course of the disease, even though these regions are responsible for HIV entry into the cell, and are important targets of the immune response, including neutralizing antibodies. The V3 domain, known to be variable in clade B and D viruses, is relatively conserved in HIV-1 subtypes A, C, G, E and H [Los Alamos website. Available:http://www.hiv.lanl.gov/content/sequence/HIV/COMPENDIUM/1999/7/V3LoopEvolution.pdf. Accessed 2014 March 31, and 9, 23, 24]. It plays a critical role in virus entry, since it binds both CCR5 and CXCR4 receptors and is a target of neutralizing antibodies [9], [23], [25], [26]. The C3-V4 region has been reported to be a major target of the early autologous neutralizing response in HIV-1 subtype C infection and the N-terminal portion of C3, which contains the α2 helix region, may play a role in virus escape processes [6], [24], [27], [28]. It has also been reported that some residues in C3 bind the CD4 receptor, in association with amino acids in the C4 region [29]–[32].

In the present study, we have therefore investigated how amino acid characteristics of the constant C3, C4 and V3 regions of HIV-1 subtype C (i.e. sequence length, number of PNGSs, number of sites under positive selection pressure and electrical charges) are modulated during the course of the disease. We used a novel integrative bioinformatics sequence analysis which allowed us to hypothesize a model to explain how conformational changes in these regions may regulate virus escape from the immune response.

## Materials and Methods

### Ethics Statement

The study was approved by the Committee for Research on Human Subjects (Medical) at the University of Witwatersrand and by the Ministry of Health and Social Welfare Scientific Ethical Committee of Swaziland. All participants provided written informed consent to participate in the study.

### Sample collection, determination of disease stage and description of the cohorts

A total of 72 plasma samples were collected from in the time period 2005 to 2007 from HIV-positive individuals, living in South Africa and Swaziland, all naïve for antiretroviral therapy (ART); 24 samples were obtained from the 2006 Swaziland HIV National Serosurvey [33], [34], 24 samples from the Chris Hani Baragwanath Hospital (CHBH) in Soweto, Johannesburg, South-Africa, in the framework of the activities included in the AVIP project (European Commission website. Available: http://ec.europa.eu/research/health/infectious-diseases/poverty-diseases/projects/84_en.htm. Accessed 2014 March 31) and 24 samples from the HIV/AIDS National Referral Laboratory (NRL) at the Government Hospital in Mbabane, Swaziland, in the framework of activities linked to the Italian AIDS Programme. Ethical clearance for these studies was obtained from local Ethical Committees.

The 72 patients were classified for disease stage into 3 groups, based on Avidity Index (AI) assay data [34], [35] and CD4+ T cell number, as previously described [9]. Briefly, by AI assay we identified those HIV infections that occurred within 6 months from blood drawing, independently of their CD4+ T cell number. A number of 24 patients were classified as recently infected (ES  =  Early disease Stage). All these patients were from Swaziland, collected in the frame of the 2006 Swaziland National Serosurvey [33], [34]. Patients that by AI were identified having an established infection were further classified into patients at chronic or late disease stage, according to their CD4+ T cell number. Specifically, 24 patients with a CD4+ T cell count >200/µl were classified in the chronic disease stage group (CS  =  Chronic disease Stage), whereas 24 patients with a CD4+ T cell count ≤200/µl were included in the late disease stage group (LS  =  Late disease Stage). CD4+ T cell number median values were 544,5/µl (values ranging from 418 to 811) and 122/µl (values ranging from 7 to 199) for CS and LS groups, respectively. The 24 patients included in the CS group were from South Africa. Samples from 6 out of these 24 patients were collected in 2005. The remaining 18 samples were collected in 2006. The 24 patients included in the LS group were from Swaziland. Samples from 13 out of these 24 patients were collected in 2006. The remaining 11 samples were collected in 2007.

### CD4+ T cell count

CD4+ T cell count was performed by means of MultiTEST and TruCOUNT tubes (Beckton Dickinson) according to the manufacturer's instruction.

### Viral RNA extraction and sequence amplification

Viral RNA was extracted from 0.5 ml of plasma using Qiamp viral RNA mini Kit (Qiagen), after treatment with Heparinase (Sigma).

The V1 to V5 coding region in the *env* gene was amplified by RT-PCR using SuperScriptTM One-Step RT-PCR System (Invitrogen) and specifically designed outer primers for clade C. The following primers were used: AC-Env Outer For  =  5′CAGATGCATGAGGATATAATCA3′; ED12 m Outer Rev  =  5′AGTGCTTCCTTGCTGCTCCCAA3′. The RT-PCR mix was composed of 0.5 to 1 µg of RNA and an RT/Taq buffer mixture containing dATP, dCTP, dTTP, dGTP 0.4 mM (Roche), MgSO_4_ 2.4 mM (Invitrogen), 1 µl RT/Taq-platinum (Invitrogen), RNase inhibitor 40 U/µl (Invitrogen), and primers 10 µM (MWB-Biotech); RT-PCR reaction was carried out using a thermal cycler (Eppendorf) and the following program: 45°C for 30′ for retro-transcription and 94°C for 2′ for the RT denaturation; the resulting cDNA was amplified as follows: 94°C for 15″, 50°C for 30″ and 68°C for 1′30″for a total of 40 cycles and a final extension step at 68°C for 7′.

The resulting PCR product was again amplified in a nested PCR, using specifically designed primers in order to obtain nucleotide sequences corresponding to the V3-C3 and the V4-C4-V5 Env regions, respectively. Primers specific for each region were the following: for the V3-C3 region: V3C3ForA  =  5′CTGTTAAATGGTAGCCTAGC3′ and V3C3RevA  =  5′GCAATAGAAAAATTCTCC3′. If amplification failed, a nested PCR was repeated using another couple of inner primers, i.e. V3C3ForB  =  5′CACAGTACAATGTACACATG3′ and V3C3RevB  =  5′RCAATAGAAAAATTCTCCTC3′; for the V4-C4-V5 region: V4-AFor5′  =  5′GTRGAGGAGAATTTTTCTATTG3′, V5-ARev5′ =  TATAATTCACTTCTCCARTTGTC. If amplification failed, a nested PCR was repeated using another couple of inner primers: V4-BFor5′  =  TTTAATTGTRGAGGAGAATTTTTCTATTG3′, V5-BRev5′ TATTTATATAATTCACTTCTCCAATTGTC3′. Each nested PCR reaction was conducted as follows: 1–5 µl aliquot of the amplified product was added to a reaction mixture containing dNTPS 200 µM (Roche), MgCl_2_ 2.5 mM (Invitrogen), the couple of 20 µM primers corresponding to the region that had to be amplified (MWB-Biotech) and AmpliTaq Gold DNA polymerase 2.5 U/µl (Applied Biosystems). Amplifications were carried out specifically for each region, as following: V3–C3: 96°C for 7′ (1st cycle), then 15″at 94°C, 30″ at 50°C, 30″ at 72°C, for 40 cycles; V4–C4–V5 region: 96°C for 7′ (1st cycle), then 15″ at 94°C, 30″ at 44°C, 30″ at 72°C, for a total of 40 cycles and a final extension step of 7′ at 68°C.

### DNA purification and sequencing

Amplified DNA of each region was purified using Qiaquick PCR purification kit (Qiagen), according to the manufacturer's protocol. The DNA samples were then quantified and checked for purity by measuring absorbance at 260 nm and 280 nm. The V3–C3 and V4–C4–V5 amplified regions were then sequenced using BigDye (Applied Biosystem), with the same primers as the nested PCR. Sequencing was performed on uncloned PCR products to identify the prevalent viral quasispecies.

The electropherogram analysis was edited using Chromas Pro (Technelysium website. Available: www.technelysium.com.au/ChromasPro.html. Accessed 2014 March 31). All sequences were aligned using ClustalW by Bioedit [36] and corrected for the multiple alignment by manual editing. The nucleotide sequences were translated into amino acid sequences using GeneRunner (www.generunner.net) and further codon-aligned using BioEdit [36].

### Phylogenetic analysis

Phylogenetic analysis was based on the V3-V5-coding region from 72 HIV-1 variants isolated from the 72 patients (one variant/patient) included in the cohorts. It was carried out using the PAUP software (version 4.0) (http://paup.csit.fsu.edu/downl.html) with the K81 model of substitution and using both Neighbor-Joining (NJ) and Maximum Likelihood (ML) treebuilding methods. The evolutionary model was chosen as the best fitting nucleotide substitution model in accordance with the results of the Hierarchical Likelihood Ratio Test (HLRT) implemented in the MODELTEST software (version 3.6) [37].

The parameters for the nucleotide substitution model were estimated by the ML method using a NJ tree (Jukes-Cantor distance) as the base tree [38].

The statistical robustness and reliability of the branching order within phylogenetic trees were confirmed by either a bootstrap analysis using 1000 replicates, for the NJ tree, or the zero branch length tests for the ML tree. All calculations were performed with PAUP software (version 4.0).

### Analysis of predicted co-receptor usage and identification of PNGSs

The analysis of predicted co-receptor usage was performed by using Geno2pheno (Genafor website. Available: http://coreceptor.geno2pheno.org/index.php. Accessed 2014 March 31) upgraded version, June 17, 2011.

Identification of PNGSs was performed by using N-GLYCOSITE (Los Alamos website. Available: http://www.hiv.lanl.gov/content/sequence/GLYCOSITE/glycosite.html. Accessed 2014 March 31).

### Analysis of electric charges

To determine the electric charge of each region, the algebraic sum of all charged residues, both positive and negative, was considered as total electric charge (Q_tot_). The sum of all positive charged residues was considered as total positive charge (Q_pos_) and the sum of all negative charged residues as total negative charge (Q_neg_).

An original Matlab (the MathWorks Inc. USA) program was written to yield the distribution of Q_tot_, Q_pos_ and Q_neg_ directly from the *.fas files with the aligned sequences. Charge distributions were analyzed with the Wilcoxon's rank sum test, after a preliminary checking of the non-Gaussian nature of the distributions using the Kolmogorov-Smirnov test, as previously described [39]. Finally, the charge correlation coefficient was calculated with Matlab.

### Codon specific dN/dS ratio

The CODEML program implemented in the PAML 3.14 software package (UCL website. Available: http://abacus.gene.ucl.ac.uk/software/paml.html. Accessed 2014 March 31) was used to investigate the adaptive evolution of the V3, C3 and C4 regions of HIV-1 *env* gene [40]. For each region, sequence alignment was performed prior to testing which amino acids were under positive selection. Six models of codon substitution, M0 (one-ratio), M1a (nearly neutral), M2a (positive selection), M3 (discrete), M7 (beta), and M8 (beta and omega) were applied in this analysis [41]. Since these models are nested, we used codon-substitution models to fit the model to the data using the likelihood ratio test (LRT) [42].

The “Bayes empirical Bayes” (BEB) approach implemented in M2a and M8 was used to determine the positively selected sites by calculating the posterior probabilities (P) of the different classes for each site [43].

Finally, the Shannon's entropy analysis was performed by using the Entropy software (Los Alamos website. Available: http://www.hiv.lanl.gov/content/sequence/ENTROPY/entropy_one.html. Accessed 2014 March 31).

The strength of positive selection (*i*) for each amino acid site was calculated as previously described [29], by defining the Weighted Mean Value (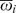) as:
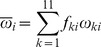
where 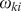 represents the k-th ω value for the i-th site, and the weight 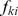 is the associated posterior probability, according to model M8 (10 values of ω are chosen for the discrete beta distribution, and another value of ω is not constrained by the beta distribution and is allowed to be greater than 1). For each pair of data set, each one associated to a disease stage, we tested whether the strength of positive selection was significantly different (H1), as opposed to being equivalent (H0), by using a paired Wilcoxon rank sum test with a continuity correction applied to the normal approximation for the *P* values [44]. Only shared sites having a weighted mean ω_i_ value greater than 1 in the two data sets being compared were included in the test.

### Three-dimensional mapping and contact maps

The core structure used for mapping the positive selected residues corresponds to the X-ray structure of CD4-bound JR-FL gp120 (Protein Data Bank [PDB] ID: 2B4C) [45]. This structure was chosen because it provides the spatial representation of V3, and is therefore useful for the identification of the PS residues in V3 (besides those in C3 and C4) in the appropriate structural context. Moreover, the chosen structure derives from a strain that has the same co-receptor usage as the majority of the isolates hereby characterized. Three-dimensional images were generated using the pyMOL v. 1.3 software (PyMOL website. Available: http://www.pymol.org/ Accessed 2014 March 31).

For generation of the contact maps, we referred to the same gp120 structure used for 3D mapping [45]. The contact map is a distance matrix that identifies, in a known 3D structure (e.g., taken from Protein Data Bank), the proximal contact between two areas. The contact distances between C-alpha carbons were used to identify interactions between regions or differences between the clades. A 10 Å cut-off distance between the C-alpha carbons was considered as a measure for contact [27]. Contact maps were generated with CMView v0.9.6, after loading the appropriate molecular structure by specifying its PDB identification number. The software allows to highlight relevant regions (e.g. C3 or C4 of Env) or single residues of the loaded structure.

### Statistical analysis

The Wilcoxon rank sum test was used to evaluate the statistical significance of the difference between parameters analysed in the study (if not explicitly stated otherwise).

Furthermore, to measure existing correlation between sequence length and glycosylation status, the Spearman's correlation test was used. All analyses were conducted using the Stata 8.2 software.

## Results

### Phylogenetic analysis

**Figure 1:**
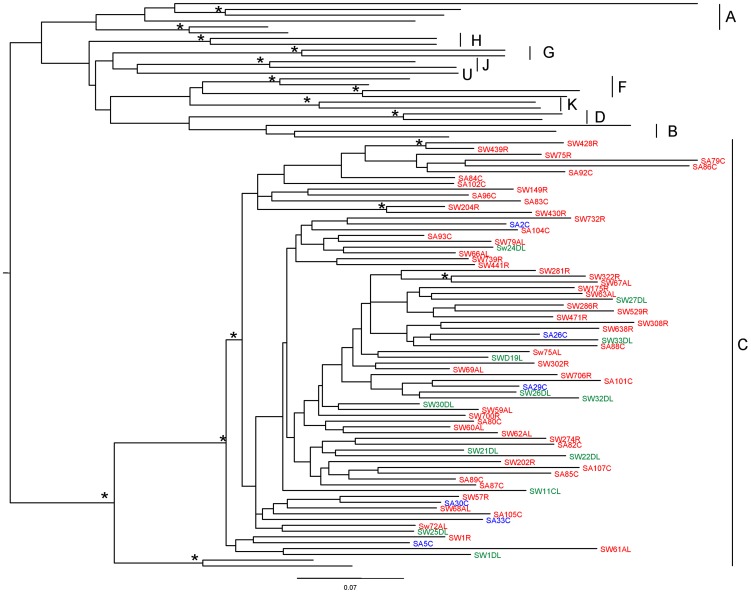
V3–V5 phylogenetic tree of HIV-1 strains from 72 HIV-1-infected patients in South Africa and Swaziland. Tree was rooted by using the midpoint rooting method. Scale bar at the bottom indicates 7% nucleotide substitutions per site. The asterisk along a branch represents the bootstrap value (significant statistical support) >70% and p<0.001 in the zero-branch-length test. Years of collection of samples are color-highlighted: 2005 in blue, 2006 in red and 2007 in green. Country where sample collection was performed: SW  =  Swaziland; SA  =  South Africa.

We amplified and sequenced the V3-V5-coding regions of HIV variants isolated from 72 HIV-infected individuals living in South Africa and Swaziland, naïve for ART during the years 2005 to 2007. A phylogenetic analysis based on the V3–V5 sequence (Fig. 1) revealed that all patients were infected by HIV-1 subtype C variants, as also previously described [9]. In the figure virus sequences from patients have been highlighted with different colors according to year of collection of sample: 2005 in blue, 2006 in red and 2007 in green. Since we wanted to study if and how sequence molecular characteristics changed during the evolution of the disease, we had to verify that the natural Env sequence evolution through the three year period would not have influenced the molecular analyses we performed. As it is evident in figure 1, the different years are well intermixed in the figure, indicating that the sampling year was not a main causal factor for the observed differences in the sequence characteristics of the Env regions we have considered for our studies. Furthermore, the phylogenetic analysis in figure 1 shows an intermixing of all the sequences, independently of the patient region of origin, indicating that a possible, if any, difference in the ethnical background of the two populations from Swaziland and South Africa does not constitute a bias for the further analysis we performed.

### Amino acid sequence length and PNGS distribution

Lengths of the amino acid sequences of the gp120 V3, C3 and C4 regions from the HIV-1 infecting virus variant obtained from each one of the 72 patients, as well as the predicted tropism of the variant, are reported in Table 1. As shown in the Table, the median length of amino acid sequences of the V3, C3 and C4 regions did not significantly vary with the disease stage. The V3 sequence length was equal to 35 amino acids in virus variants from 66 out of 72 samples, 34 amino acids in 5 variants, and 37 amino acids in 1 variant. In the variants with a V3 sequence of either 34 or 37 amino acids, no correlation with disease stage was found. In fact, of the five variants with a V3 sequence of 34 amino acids, 2 were from ES, 2 from CS and 1 from LS patients. The only variant with a V3 sequence of 37 amino acids was obtained from an LS patient. Regarding the predicted viral tropism, the three sample groups presented a similar pattern with 20 out of 24 variants being R5 tropic and 4 being X4 tropic in the ES group; 19 R5 and 5 X4 tropic in the CS group and 18 R5 tropic and 6 X4 tropic in the LS group.

**Table 1:** Amino acid sequence length of C3, C4 and V3 regions and estimated tropism of HIV-1 clade C variants from patients at different disease stages.

	ES patients (1 to 24)				CS patients (25 to 48)				LS patients (49 to 72)			
				Tropism				Tropism				Tropism
	Sequence length (n. of aa)				Sequence length (n. of aa)				Sequence length (n. of aa)			
	52	41	35	R5	52	41	35	R5	53	41	35	R5
	52	41	35	R5	52	41	35	R5	52	41	35	R5
	50	41	35	R5	52	41	35	R5	52	41	35	R5
	53	41	35	R5	52	41	35	R5	52	41	35	R5
	52	41	35	R5	52	41	35	X4	52	41	35	R5
	53	41	35	R5	52	41	35	R5	52	41	35	R5
	51	41	35	R5	52	41	34	X4	52	41	35	X4
	52	41	35	R5	52	41	35	X4	52	41	35	R5
	52	41	35	X4	52	41	35	R5	52	41	35	R5
	52	41	35	R5	52	41	34	R5	52	41	35	R5
	54	41	35	X4	52	41	35	R5	52	41	35	X4
	50	41	35	R5	52	41	35	R5	52	41	34	R5
	52	41	35	R5	52	41	35	X4	52	41	35	X4
	53	41	35	X4	52	41	35	R5	52	41	35	R5
	52	41	35	R5	52	41	35	X4	52	41	35	R5
	51	41	35	R5	52	41	35	R5	52	41	35	X4
	52	41	35	R5	51	41	35	R5	52	41	35	R5
	52	41	35	R5	52	41	35	R5	52	41	35	R5
	53	41	35	R5	52	41	35	R5	53	41	35	X4
	52	41	35	R5	52	41	35	R5	52	41	35	R5
	52	41	35	R5	51	41	35	R5	52	41	35	R5
	52	41	34	R5	51	41	35	R5	51	41	37	X4
	52	41	35	X4	52	41	35	R5	52	41	35	R5
	52	41	34	R5	52	41	35	R5	52	41	35	R5
**Median n. of aa (range)**	**52 (50–54)**	**41 (41**–**41)**	**35 (34**–**35)**		**52 (51**–**52)**	**41 (41**–**41)**	**35 (34**–**35)**		**52 (51**–**53**	**41 (41**–**41)**	**35 (34**–**37)**	

**ES  =  Early disease Stage; CS  =  Chronic disease Stage; LS  =  Late disease Stage; aa  =  amino acids.**

Amino acid sequence length of the C3 region ranged from 50 to 54 amino acids. No significant differences in sequence length among the disease stages were observed (Table 1), although a tendency to a broader length variability was detected in the ES group where 15 variants had a C3 region of 52 amino acids, 4 of 53, 2 of 51, 2 of 50 and 1 of 54. In the CS and LS groups, the C3 region was more conserved with 21 variants bearing a C3 region of 52 amino acids in both disease stages. The remaining 3 variants in the CS group had a C3 region of 51 amino acids, whereas in the LS group, 2 had a C3 region of 53 amino acids and 1 of 51 amino acids. The C4 region length was of 41 amino acids in all 72 variants.

**Figure 2:**
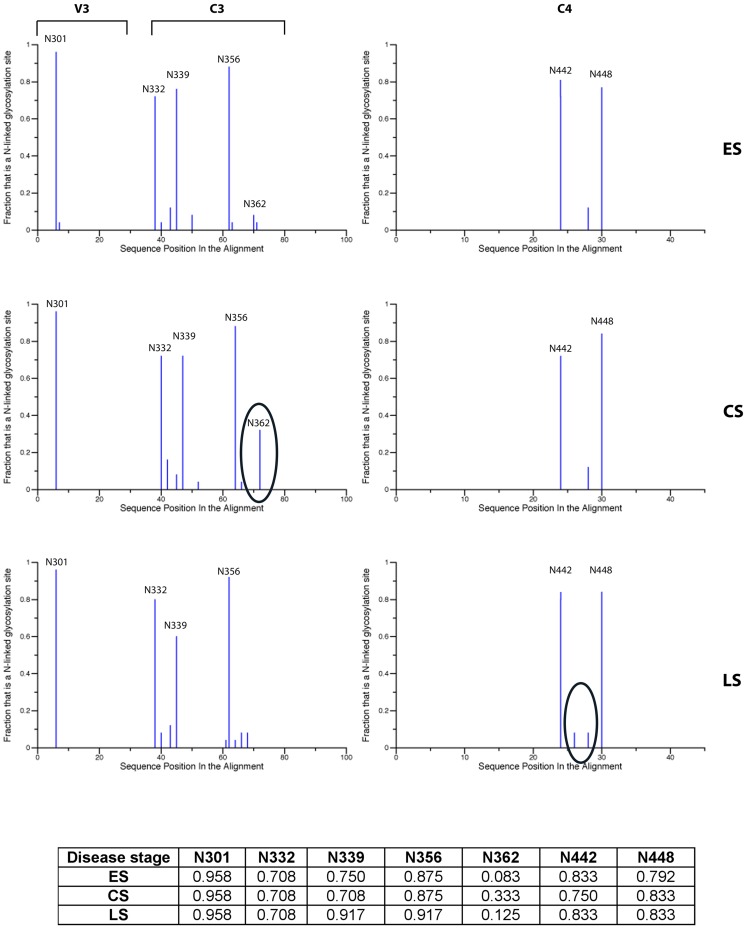
Distribution of PNGS in V3, C3 and C4 regions of gp120 in HIV-1 clade C variants obtained during different disease stages. Table at the bottom reports the frequency of presence of each PNGS. PNGS position in the alignment is according to the HXB2 amino acid sequence numbering. Circles highlight a significant increase of *shifting* PNGS frequency ES  =  Early disease Stage CS  =  Chronic disease Stage LS  =  Late disease Stage.

Table 2 reports the mean and median number of PNGSs for each region and for each disease stage. Overall, both the median and the mean number of PNGSs of each region did not show statistically significant variations among the three disease stages. A qualitative analysis of the distribution of PNGSs among the different groups of patients is reported in figure 2. A number of PNGSs, in particular, PNGS N301 (in V3), N332, N339 and N356 (in C3), and N442 and N448 (in C4) are conserved in variants obtained from patients at all the disease stages with a frequency higher than 70%, with the exception of PNGS 339 (numbering according to HXB2 sequence) in the C3 region that is present in virus variants obtained from patients at the LS stage where it is represented with a frequency of about 60%. For this reason these PNGSs have been named “*fixed*”. Besides these, other PNGSs were found with a lower frequency in all the regions. Most of these PNGSs are represented with a frequency that is often <20% and are not constantly present in virus variants obtained from patients at all disease stages. Therefore, they have been named “*shifting*” PNGSs, in agreement with previous reports [9], [14], [46], [47]. The majority of the *shifting* PNGSs are present in the C3 region in variants obtained during the chronic stage of the disease, in particular in the α2 helix (amino acids 335 to 352) and in the N-terminal and in C-terminal portions of the C3 region. The PNGS at position 362 was found with a frequency of 33.3% in the variants from the CS group (encircled in the figure), whereas it is present at much lower frequency in variants from the ES and LS groups. A *shifting* PNGS between N442 and N448 in the C4 region was detected only during the late stage of the disease (encircled in the figure). Of note, we also found a low frequency of expression, at all disease stages, of the glycosylated residue 295, which has been described to be highly conserved in other HIV-1 subtypes [47] that comprehends the last amino acid in the C2 region and the first two in the V3 region. Since this PNGS is located between the C2 and the V3 regions, it has not been reported in figure 2.

**Table 2:** Number of PNGSs in gp120 V3, C3 and C4 regions of HIV-1 clade C variants from patients at different disease stages.

		Number of PNGSs		
Disease stage		C3	C4	V3
**ES**	**Mean**	2.75	1.71	1.00
	**Median**	3.00	2.00	1.00
	**Range**	2–4	0–2	0–2
**CS**	**Mean**	2.96	1.71	0.96
	**Median**	3.00	2.00	1.00
	**Range**	1–4	1–2	0–1
**LS**	**Mean**	2.67	1.83	0.96
	**Median**	3.00	2.00	1.00
	**Range**	2–4	1–2	0–1

**ES  =  Early disease Stage; CS  =  Chronic disease Stage; LS  =  Late disease Stage.**

### Analysis of positive selection

For each disease stage, we evaluated which amino acid residue in the constant V3, C3 and C4 regions was under positive selection pressure, by calculating the dN/dS ratio for each position. Table 3 lists the sites that have a mean dN/dS ratio >1 in variants from at least one of the disease stages. The number of amino acid positions under positive selection pressure is considerably higher in the C3 region at the chronic disease stage. In fact, in this region, ten, twelve and three residues appear to be under positive selection in the virus variants from ES, CS and LS groups, respectively. Nine out of twelve residues under positive selection pressure in the variants from CS group cluster in the N-terminal portion of the C3 domain, within the α2 helix (amino acids 335 to 352). Within the V3 domain, only three sites are under positive selection pressure (amino acids 300, 309 and 322) in variants from the CS group, two in variants from the ES group (amino acids 300 and 322) and two in variants from the LS group (amino acids 300 and 309). In the C4 domain only one site is under positive selection pressure (amino acid 429) in virus variants obtained at all disease stages. In this region, an additional amino acid at position 446 appears to be under positive selection pressure only in the LS group.

**Table 3:** Amino acid sites under positive selection in gp120 V3, C3 and C4 regions of HIV-1 clade C variants from patients at different disease stages.

			Mean dN/dS ratio		
Region	Position in HXB2	Position in JR-FL	ES	CS	LS
**V3**	300	297	3.056±0.699	3.056±0.690	4.676+1.223
	309	306	<1	3.019±0.718	4.676+1.226
	322	317	3.050±0.707	3.050±0.696	<1
**C3**	335	331	4.711±0.995	3.507±0.122	<1
	336	332	4.902±0.598	3.507±0.118	7.808±1.550
	337	333	4.864±0.703	3.505±0.138	<1
	343	339	4.902±0.599	3.507±0.120	<1
	344	340	4.901±0.600	3.507±0.118	<1
	346	342	4.902±0.599	3.507±0.118	<1
	347	343	<1	3.505±0.143	<1
	350	346	4.885±0.648	3.507±0.118	<1
	352	348	<1	3.494±0.220	<1
	360	355	4.902±0.598	3.504±0.145	<1
	362	357	4.902±0.598	3.507±0.118	7.807±1.552
	364	359	4.902±0.598	3.507±0.120	7.808±1.550
**C4**	429	420	2.935±0.911	5.321±1.659	2.905±0.591
	446	437	<1	<1	2.893±0.604

**ES  =  Early disease Stage; CS  =  Chronic disease Stage; LS  =  Late disease Stage.**

**Figure 3:**
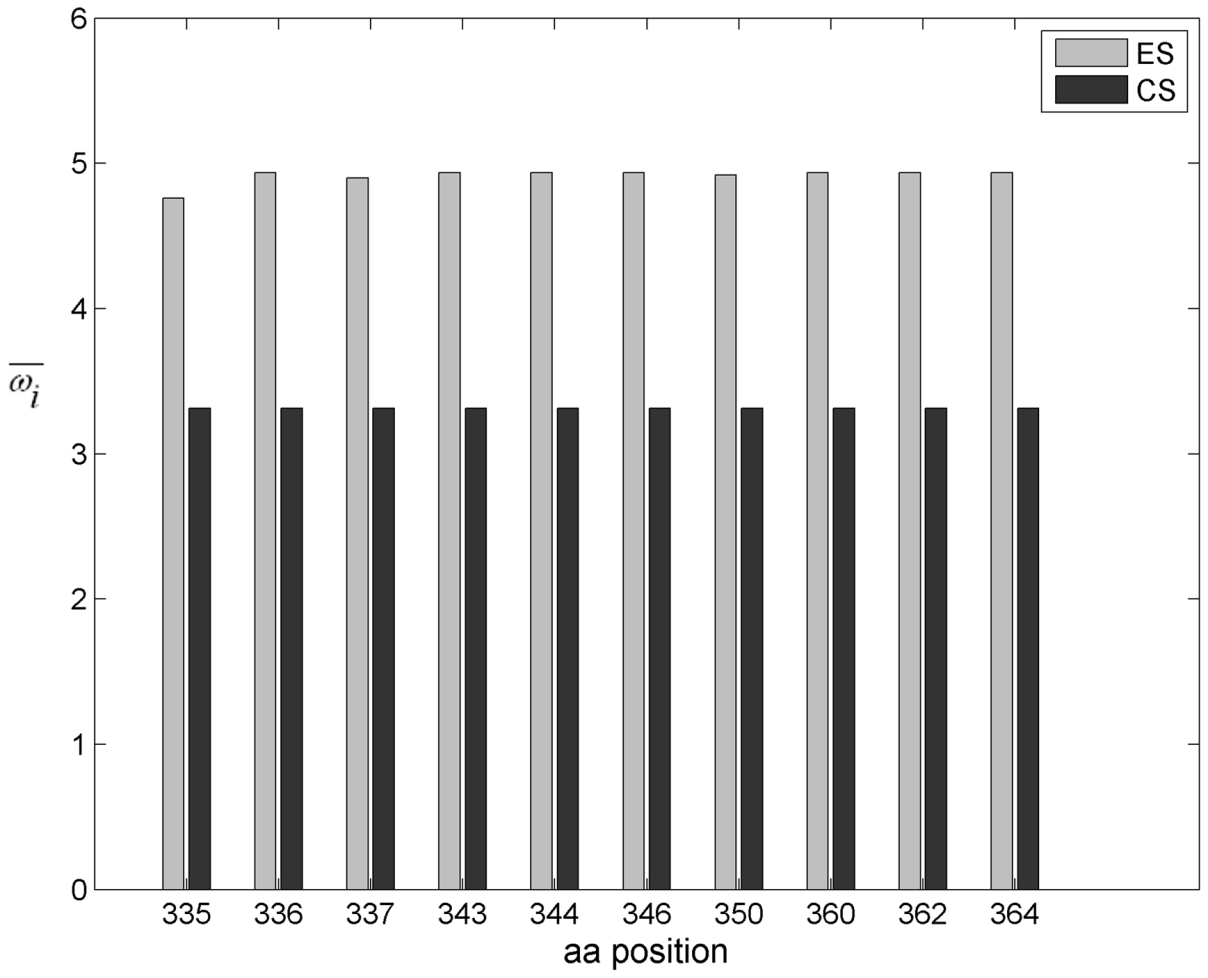
Strength of positive selection of sites in the C3 region during the early and chronic disease stages. The strength of positive selection (ω) for each amino acid site is reported in the y axis. Sites are reported in the x axis. Amino acids numbering is according to HXB2 sequence. Grey and black bars show the strength of positive selection of each of the sites for the early infection stage (ES) and the chronic infection stage (CS), respectively. Statistical significance of the difference between the two series: p = 0.00016.

We have then calculated the strength of the positive selection pressure for each one of the identified residues. No statistically significant difference between the set of weighted mean values (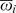) among the disease groups was found for both the V3 and C4 regions (data not shown). Conversely, for the C3 domain, a significant difference (p = 0.00016) was found between the set of weighted 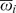 of the ES and CS groups (Fig. 3), with sequences from variants of the ES group displaying residues with higher 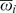 values than the ones calculated for the CS group.

### Molecular visualization of sites under positive selection pressure

**Figure 4:**
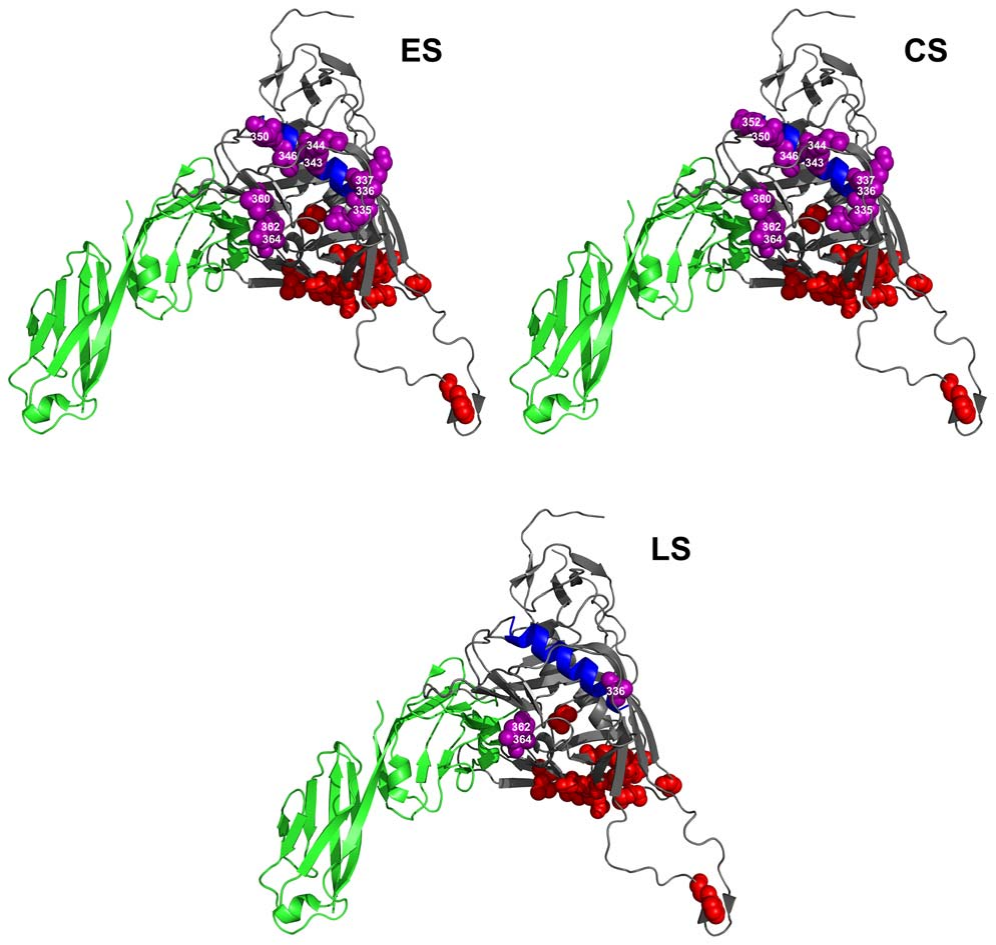
Sites under positive selection pressure in C3 region at different disease stages. HIV-1 gp120 protein model is from JR-FL strain [45]. Sites under selection pressure are indicated as spheres in purple. Labeling of each positively selected site is centered on the α-carbon of the amino acid. CD4 receptor is in green. Residues involved in CCR5 binding are indicated as spheres in red. The α2 helix region is indicated in blue. Amino acid numbering is according to HXB2 sequence. ES  =  Early disease Stage CS  =  Chronic disease Stage LS  =  Late disease Stage.

**Figure 5:**
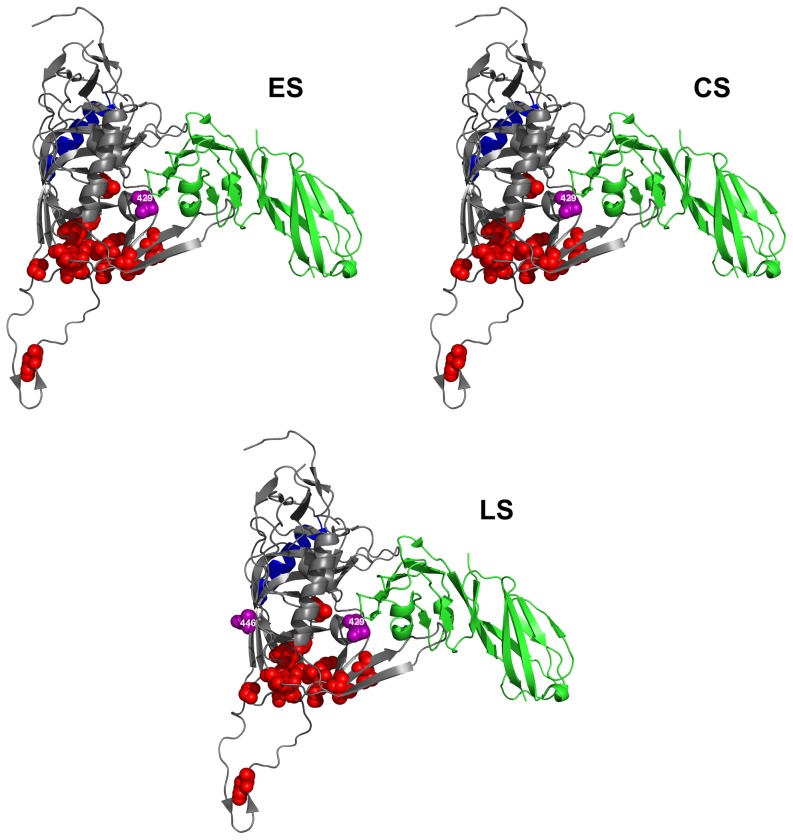
Sites under positive selection pressure in C4 region at different disease stages. HIV-1 gp120 protein model is from JR-FL strain [45]. Sites under selection pressure are indicated as spheres in purple. Labeling of each positively selected site is centered on the α-carbon of the amino acid. CD4 receptor is in green. Residues involved in CCR5 binding are indicated as spheres in red. The α2 helix region is indicated in blue. Amino acid numbering is according to HXB2 sequence. ES  =  Early disease Stage CS  =  Chronic disease Stage LS  =  Late disease Stage.

**Figure 6:**
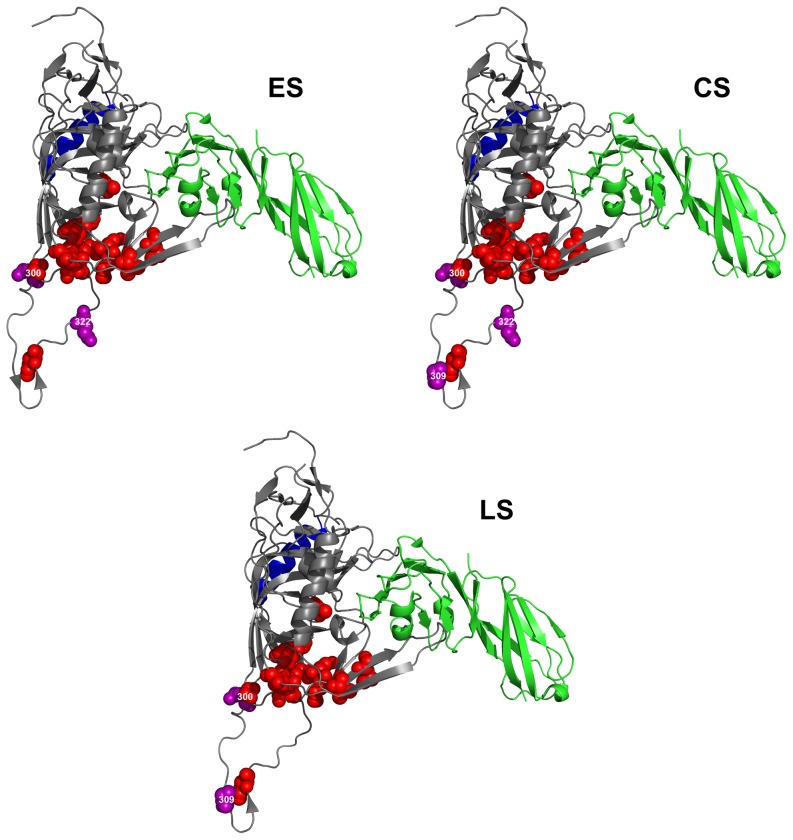
Sites under positive selection pressure in V3 region at different disease stages. HIV-1 gp120 protein model is from JR-FL strain [45]. Sites under selection pressure are indicated as spheres in purple. Labeling of each positively selected site is centered on the α-carbon of the amino acid. CD4 receptor is in green. Residues involved in CCR5 binding are indicated as spheres in red. The α2 helix region is indicated in blue. Amino acid residue 300 in V3 is partially hidden by residue 441 of the C4 region, to which it is spatially close. Amino acid numbering is according to HXB2 sequence. ES  =  Early disease Stage CS  =  Chronic disease Stage LS  =  Late disease Stage.

In order to characterize the positions of the residues under positive selection in the gp120 protein, we mapped them on the three-dimensional structure of the CD4-bound gp120 derived from the HIV-1 JR-FL strain [45]. Figures 4, 5 and 6 show the positions of the residues under positive selection in the C3, C4 and V3 regions (spheres in purple), respectively. Sites in C3 from variants obtained from the ES and CS groups are more numerous than those found in the variants from the LS group (Fig. 4). These sites are located in the α2 helix region (in blue), near the CD4-binding site and close to residues critical for the binding to CCR5 (spheres in red). Residues within the C3 region that are critical for the binding to CD4 (amino acids 366 and 368, not shown in the figure, since hidden by the CD4 structure) are fully conserved in virus variants obtained at all the three disease stages, as also confirmed by the low Shannon Entropy values (data not shown). In contrast, the number of sites under positive selection pressure in the C4 region (Fig. 5) is small in the virus variants from all the three disease groups. Again, these residues are placed near the CD4-binding site or close to residues critical for CCR5 binding. Finally, the few sites under positive selection in the V3 region are all proximal to the highly conserved residues critical for CCR5 binding (Fig. 6).

### Electric charge analysis and interaction between regions

**Figure 7:**
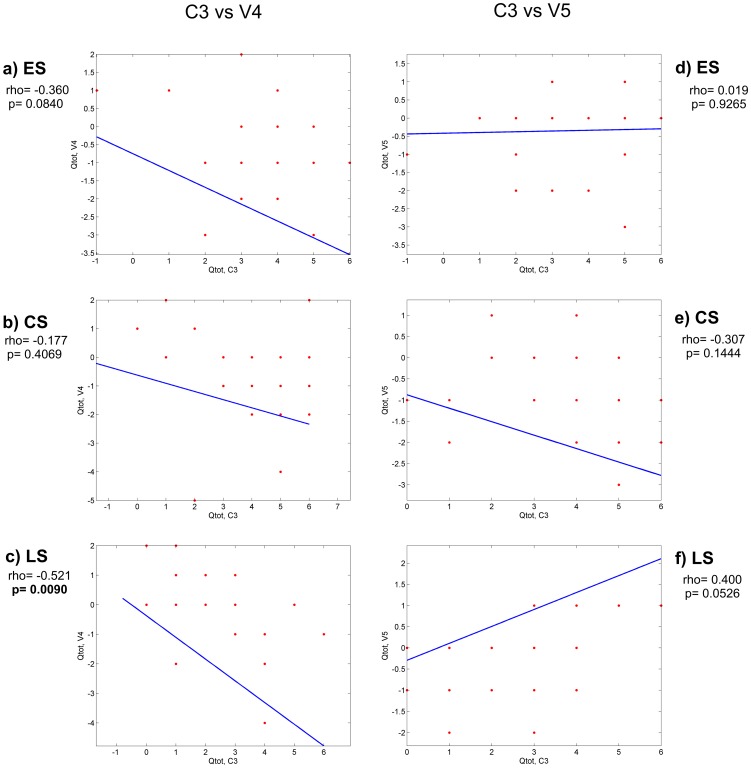
Electric charge correlations between C3-V4 and C3-V5 regions. Statistically significant p values are in bold. Rho  =  correlation coefficient ES  =  Early disease Stage CS  =  Chronic disease Stage LS  =  Late disease Stage.

**Figure 8:**
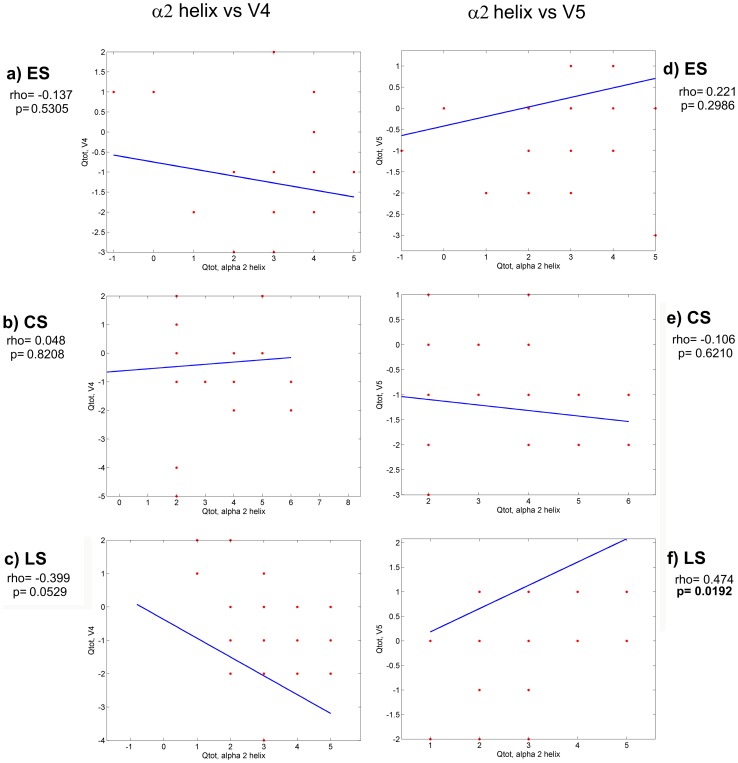
Electric charge correlations between α2 helix-V4 and α2 helix-V5 regions. Statistically significant p values are in bold. Rho  =  correlation coefficient ES  =  Early disease Stage CS  =  Chronic disease Stage LS  =  Late disease Stage.

In order to investigate whether the electric charges of C3 and C4 regions of gp120 (that are the principal components of the CD4 binding site) can have an impact on the conformation of the CD4 binding site, we calculated the electric charge of each one of these regions and correlated it with the electric charges of all the other variable regions. After computing all the total electric charge (Q_tot_) correlations, we found statistically significant correlations between C3 and V4 and between C3 and V5 regions. These correlations are shown in figures 7 and 8. A negative correlation was present in the C3-V4 interaction (increased Q_tot_ in V4 region and decreased Q_tot_ in C3 region) at all stages of disease (Fig. 7). This correlation was statistically significant in the LS group of patients (panel c, p = 0.0090). The electric charge analysis of the C3-V5 interaction showed a strong tendency to a positive correlation (increased Q_tot_ in both C3 and V5) in the virus variants from LS group of patients (panel f, p = 0.0526). This correlation was statistically significant when the interaction between electric charges of α2 helix (in C3 region) and V5 was considered (Fig. 8 panel f, p = 0.0192).

**Figure 9:**
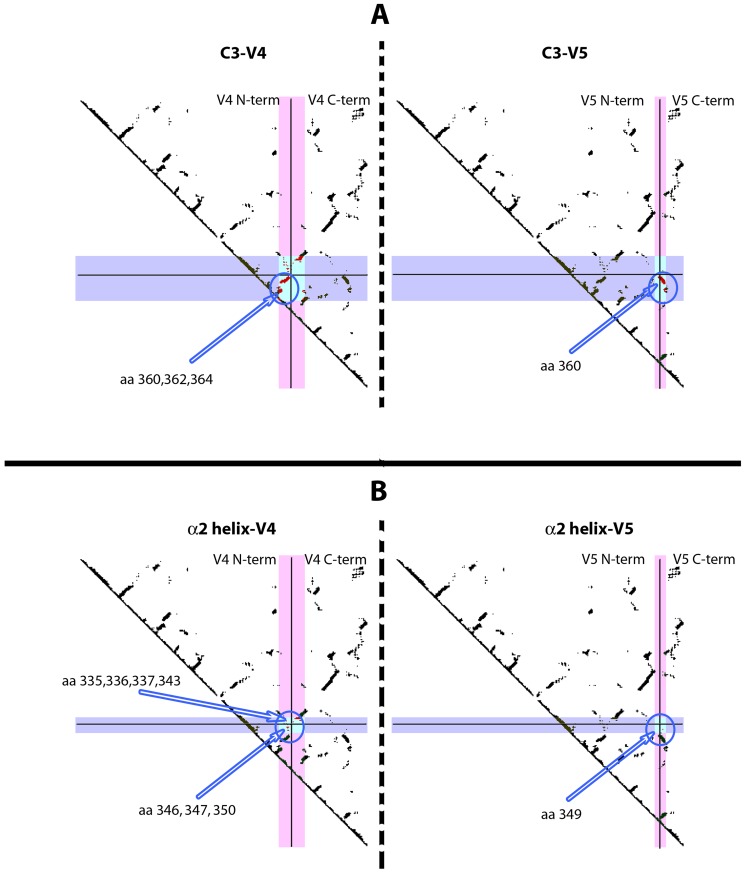
Contact maps between C3-V4, C3-V5, α2 helix-V4 and α2 helix-V5 of gp120. Contact maps are generated from the crystallographic structure of the CD4-bound JR-FL2 gp120 [45]. Regions are indicated in violet and in pink. The boxed area in light blue is the contact area. Solid lines split the region in the N-terminal and C-terminal portions. In the contact areas, only amino acids that have been found under positive selection are shown (indicated by arrows and circled). Amino acid numbering is according to HXB2 sequence. Panel A) left: C3 region is reported horizontally (in violet), V4 region vertically (in pink). Panel A) right: C3 region is reported horizontally (in violet), V5 region vertically (in pink). Panel B) left: α2 helix is reported horizontally (in violet), V4 region vertically (in pink). Panel B) right: α2 helix is reported horizontally (in violet), V5 region vertically (in pink).

The correlations of the electric charges between C3 and V4, C3 and V5, α2 helix and V4, and α2 helix and V5 were then further characterised by means of contact maps, which allowed us to identify close spatial contacts between two regions. Figure 9 shows the contact maps for C3-V4 and C3-V5 (Panel A), and α2 helix-V4 and α2 helix-V5 (panel B) in the crystallographic structure of the JR-FL gp120 bound to CD4 [45]. As shown in panel A (top), C3 and V4, and C3 and V5 regions strongly interact (light blue area). In particular, amino acids in C3 at positions 360, 362 and 364, which have been found under positive selection pressure in the virus variants obtained during the chronic disease stage, interact with N-terminal residues of V4 region (panel A, left). In the interaction between C3 and V5 (panel A, right) a single residue at position 360 of the C3 region, under positive selection pressure in the variants from the chronic disease stage, was found to interact with residues in the C-terminus of the V5 region.

In the panel B of figure 9, the interactions of α2 helix with V4 and V5 regions are reported. Residues 335, 336, 337, 343, 346, 347 and 350 in α2 helix, which have been found under positive selection pressure in the chronic disease stage, interact with residues in the C-terminal and N-terminal of V4 (left). Finally, a single residue in the α2 helix, at position 349, interacts with one residue in the C-terminal of the V5 region (right).

## Discussion

The role of gp120 variable regions in HIV escape from immune response has been previously investigated (9–17). However, the role of constant regions of gp120 has not been thoroughly studied to date. In the present work we focused on those gp120 constant regions of HIV-1 clade C which are key in the binding to CD4 receptor and coreceptors and are involved in the processes of HIV-1 cell entry and in host immune response. Amino acid sequence lengths, PNGSs distribution, residues under positive selection pressure and electric charges within the V3, C3 and C4 conserved domains of HIV-1 clade C variants obtained from individuals at different stages of disease, were investigated. The aim of the study was to evaluate if and how these sequence characteristics in these constant regions were modulated during the course of the disease.

The study was performed on only patients naïve for ART to avoid influence of antiretroviral therapy on variants selection. It therefore provides a clearer picture of mechanisms of HIV immune escaping in patients who are not treated with antiretroviral drugs, a condition that, unfortunately, is still common in many poor developing countries where HIV is highly endemic.

We observed that the V3 sequence length is well conserved in all the disease stages (35 amino acids, on average), as previously reported [Los Alamos website. Available: http://www.hiv.lanl.gov/content/sequence/HIV/COMPENDIUM/1999/7/V3LoopEvolution.pdf. Accessed 2014 March 31, and 9, 23, 24], since this length possibly ensures the optimal conformation for the binding to CCR5 [48]. Virus tropism, predicted on the basis of the V3 sequence, does not switch from R5 to X4 at the later disease stage, as it has been instead described for the HIV-1 B subtype [25], [48]–[51], although a greater, but not significant, number of X4 strains have been observed at the late stage, when compared to early and chronic stages.

The amino acid length of the C3 region ranged from 50 to 54 residues. In line with previous studies, we confirmed that the C3 domain in subtype C variants is relatively variable, in particular in its α2 helix portion, although sequence length variability among disease stages was not statistically significant. The α2 helix sub-domain is more exposed to the solvent and to neutralizing antibodies in clade C than in clade B virus and thus exhibits a higher sequence entropy at the polar face [24]. Conversely, the C4 region was constant in its amino acid sequence length (41 aa) at all the disease stages. This may be due to the fact that, although located together with the C3 region in the outer domain of gp120 protein, C4 is buried into a hydrophobic pocket and therefore is less exposed to the selection pressure of the host immune response.

The number of conserved PNGSs in the V3, C3 and C4 regions was constant at all the disease stages. All conserved PNGSs in these regions are found in all M group viruses with a frequency >70% [52]. A single *fixed* PNGS in V3, at position 301, involved in coreceptor binding, was observed in the N-terminal portion [53]. No shifting PNGSs in V3 were observed at all disease stages, in agreement with previous data indicating that in the V3 domain the presence of *shifting* PNGSs is very rare, since appearance of new PNGSs may interfere with co-receptor binding [10]. In addition, we found a low frequency of expression of the glycosylated residue 295, located at the confluence of C2 and V3, within the target epitope of the 2G12 monoclonal antibody. Absence of the 295 residue in the clade C variants explains the resistance of subtype C strains to the neutralising effects of this monoclonal antibody [54]. In the C3 domain, *shifting* PNGS were mainly observed within the α2 helix and the C-terminal portions, downstream of the conserved PNGS N356. These amino acid positions are important targets of neutralizing antibodies and the generation of new PNGSs could provide a shield against the immune system response. Interestingly, we have identified a PNGS at position 362 with a frequency of 33.3% in the CS group and with a much lower frequency (8.3%) in the ES and LS groups. As observed by Sterjovski et al., this residue, adjacent to the CD4-binding site, is associated with an enhanced virus fusogenic capacity and entry [55]. Since an increase in length of the amino acid sequence of V1 and V4 regions during the chronic stage of the disease may result in a reduced affinity to the CD4 receptor [9], [56], we can speculate that the PNGS 362 in C3 may be selected during the chronic stage to compensate the reduced affinity to CD4.

In the C4 region, two conserved PNGSs at positions 442 and 448 and a few *shifting* PNGSs between these two residues, have been identified. The N442 residue was suggested to be involved in glycan shielding [57], [58], whereas the N448 residue is known to be critical for the binding to CCR3 co-receptor, together with residue N356 in C3 [59]. The N448 residue has also been reported to protect the V3 loop from neutralizing antibodies and to promote processing and/or presentation of T-helper epitopes for antiviral T-cell response [60], [61]. Since PNGSs in the C4 region are positioned close to the pocket of the CD4-binding site, the *shifting* PNGSs lying in that region may shield the virus from both neutralizing antibodies and the T-helper mediated immune response [14].

In an attempt to characterize other sites that could be involved in virus escaping, we identified those sites in V3, C3 and C4 that are under positive selection pressure at the different disease stages and mapped them onto the three-dimensional structure of the CD4-bound gp120 from the JR-FL HIV-1 virus [45]. Most of these sites under positive selection pressure were found within the C3 region and, in particular, in the α2 helix portion during early and chronic disease stages. We also detected three sites in the V3 region under positive selection pressure during the chronic disease stage, and two sites in both the early and late disease stages. The presence of sites under positive selection pressure in the α2 helix region, as well as in the V3 region, indicates a strong diversifying selection of these regions, in a subtype-specific manner [19], [29], [30], [62].

The presence of sites under positive selection pressure in C3 region of HIV-1 clade C subtype during the chronic disease stage has been described in literature [63]. However, we also found a strong reduction of sites under positive selection pressure in the C3 region during the late disease stage. This is the first time, to our knowledge, that such an evidence is reported. Lastly, in the C4 region close to the CD4 binding site, two sites were found under positive selection pressure during the late stage of the disease and only one in the early and chronic disease stages. Of note, some of the sites under positive selection pressure were also found close to critical residues for binding to either CD4 or CCR5.

The strength of positive selection was found to be significantly different among the disease stages only in the C3 region. In particular, it was found to be significantly higher at the early stage of the disease, when compared to the one at the chronic stage. Taken together these data lead us to speculate that during the early disease stage, when the immune response may be less developed compared to the chronic stage, more degrees of freedom for the three-dimensional structure of the viral particle surface are allowed. Conversely, during the chronic disease stage, the greater pressure generated by a more developed immune response may lead to the selection of more non-synonymous substitutions that are compatible with an increasingly constrained three-dimensional conformation of the gp120 molecule in terms of viral fitness, since fewer degrees of freedom are allowed. The greater number of positive selected sites in the C3 region during the chronic disease stage, in comparison to those observed during the early stage, may be seen as a compensatory effect by which the virus counteracts the lower strength of positive selection in the CS stage with an increased number of positive selected sites in this stage. This effect may, in turn, benefit a higher resistance of the virus to the immune response, in particular to the action of neutralising antibodies.

Since no significant differences in the number and the strength of positive selection were observed in both V3 and C4 regions at any disease stage, we propose that the C3 region could be a more critical target for positive selection than C4 and V3, as already suggested by Moore et al., using HIV-1 clade C chimera viruses [6]. In this regard, data in literature indicate that the α2 helix in HIV-1 subtype C is more exposed to the solvent than in HIV-1 subtype B and that the residues with dN/dS >1 are associated with resistance to neutralization [24]. Electrical charge analysis revealed a significant strong negative correlation between the total electric charges of C3 and V4 in virus variants during the late stage of disease coupled with a strong positive correlation between the total electric charges of C3 (in particular the α2 helix) and V5 during the late stage of the disease. Contact maps analysis showed that the N-terminus of the α2 helix and the C-terminus of the V4 region are in close proximity. This may be related to the charge negative correlation between C3 and V4 (and α2 helix and V4), seen in the variants at late disease stage, in agreement with previously published data [24]. Similarly, C3, in particular the α2 helix, strongly interacts with V5, with a positive electric charge interaction. Overall, data on electric charge and interaction among regions suggest that during the course of the disease changes in electric charge and strong physical interactions can affect the conformation of the regions of gp120. These changes may be instrumental in the virus escape mechanism. In fact, the negative value of the charge cross-correlation between C3 and V4 in the late disease stage may indicate that an attractive force is mediated by such changes, thus maintaining a more compact and closed structure of the two regions. Contact maps indicate that C3 and V4 are in close proximity and show a spatial preference for charged residues, suggesting that the negative correlation between C3 and V4 can have a physical explanation in terms of electrostatic interactions between these two regions. It could be assumed that the negative C3-V4 correlation is the hallmark of an electrostatic attraction between these two regions. This, in turn, induces a conformational change in both regions towards a more closed conformation that may protect the C3-V4 epitope from neutralizing antibodies. The absence of a significant C3-V4 negative correlation during the chronic disease stage could be compensated by the presence of the PNGS at position 362 in the C-terminal region of C3, whose frequency is increased during chronic disease stage. The PNGS at position 362 is near the CD4 binding site, and strongly interacts with the V4 region, as shown by the contact maps. The presence of the glycan residue at position 362 could, therefore, act as a shield against neutralizing antibodies. In late disease, the electric charge-mediated attraction between C3 and V4, which favors a more closed conformation, could be counter-balanced by the repulsive forces between C3 (in particular the α2 helix) and V5, shown here. These repulsive forces could be responsible for a more open conformation of these two regions. Since the orientation of V5 regulates the opening of the CD4 binding pocket [55 and Cenci and D'Avenio, personal communication], these data support the hypothesis that, when the immune system becomes weaker with disease progression, a more open conformation in these two regions may increase the fitness for the binding to the CD4 cell receptor, as critical residues for CD4 binding have been shown to be present in C3, in addition to those present in C4 [30], [31].

It is also noteworthy that some sites under positive selection pressure are found to be involved in the interactions between C3 and V4 (amino acids 360, 362 and 364 in C3) and between C3 and V5 (amino acids 360 and 362 in C3), hinting at a possible role in conformational changes driven by the immune pressure. Similarly, the contact map between the α2 helix and the V4 region indicates that the sites under positive selection pressure within the α2 helix (residues 335, 336, 337, 343, 346, 347 and 350) strongly interact with C terminal residues in V4 and with one residue at V4 N-terminal. It is plausible that the α2 helix structure, interacting with V4 through electric charge rearrangements, could relocate the neutralizing epitope in the C3-V4 region, thereby eluding the host's neutralizing antibodies.

## Conclusions

We have identified, for the first time and in a large sample of virus variants, several distinguishing features in V3, C3 and C4 regions of HIV clade C gp120 protein. We have described the variability of these features during the course of the disease and speculated on possible implications in virus infectivity and resistance to the host's immune response. In particular, we described the presence of sites in C3, C4 and V3 regions that are under positive selection pressure and are proximal to residues important for the binding to CD4 receptor or co-receptors, and mapped them in critical areas of contact between these regions. These sites may be instrumental to charge-associated conformational changes that may play a role in the evolution of HIV.

These findings may contribute significantly to the search for new immunogens suitable for developing a sterilising vaccine against HIV, as well as to the design of novel antiviral drugs.

**Accession codes.** Sequences included in this paper have been deposited in GenBank under the accession numbers JN121046 to JN121117.

## References

[1] Starcich BB, Hahn BH, Shaw GM, McNeely PD, Modrow S, et al (1986) Identification and characterization of conserved and variable regions in the envelope gene of HTLV-III/LAV, the retrovirus of AIDS. Cell 45: 637–648. 2423250 10.1016/0092-8674(86)90778-6

[2] Curlin ME, Zioni R, Hawes SE, Liu Y, Deng W, et al (2010) HIV-1 envelope subregion length variation during disease progression. Plos Pathog 6: e1001228. 21187897 10.1371/journal.ppat.1001228 PMC3002983

[3] Perelson AS, Neumann AU, Markowitz M, Leonard JM, Ho DD (1996) HIV-1 dynamics in vivo: virion clearance rate, infected cell life-span, and viral generation time. Science 271: 1582–1586. 8599114 10.1126/science.271.5255.1582

[4] Coffin JM (1995) HIV Population Dynamics in Vivo: Implications for Genetic Variation, Pathogenesis, and Therapy. Science 267: 485–489. 10.1126/science.7824947 7824947

[5] Pollakis G, Kang S, Kliphuis A, Chalaby MI, Goudsmit J, et al (2001) N-linked glycosylation of the HIV type-1 gp120 envelope glycoprotein as a major determinant of CCR5 and CXCR4 coreceptor utilization. J Biol Chem 276: 13433–13441. 11278567 10.1074/jbc.M009779200

[6] Moore, P.L. Ranchobe N, Moore, P.L. Ranchobe N, Lambson BE, Gray ES, Cave E, et al (2009) Limited neutralizing antibody specificities drive neutralization escape in early HIV-1 subtype C infection. Plos Pathog 5: e1000598. 19763271 10.1371/journal.ppat.1000598 PMC2742164

[7] Goudsmit J, Back NK, Nara PL (1991) Genomic diversity and antigenic variation of HIV-1: links between pathogenesis, epidemiology and vaccine development. FASEB J 5: 2427–2436. 2065891 10.1096/fasebj.5.10.2065891

[8] Letvin NL, Walker BD (2003) Immunopathogenesis and immunotherapy in AIDS virus infections. Nat Med 9: 861–866. 12835706 10.1038/nm0703-861

[9] Cenci A, Tavoschi L, D' Avenio G, Narino P, Becattini S, et al.. (2012) Characterization of variable regions of the gp120 protein from HIV-1 subtype C virus variants obtained from individuals at different disease stages in Sub-Saharan Africa. J AIDS Clin Res S8–006.

[10] Rong, R. Bibollet-Ruche F, Mulenga J, Allen S, Blackwell JL, et al (2007) Role of V1V2 and other human immunodeficiency virus type 1 envelope domains in resistance to autologous neutralization during clade C infection. J Virol 81: 1350–1359. 17079307 10.1128/JVI.01839-06 PMC1797511

[11] Kwong PD, Wyatt R, Robinson J, Sweet RW, Sodroski J, et al (1998) Structure of an HIV gp120 envelope glycoprotein in complex with the CD4 receptor and a neutralizing human antibody. Nature 393: 648–659. 9641677 10.1038/31405 PMC5629912

[12] Wyatt R, Moore J, Accola M, Desjardin E, Robinson J, et al (1995) Involvement of the V1/V2 variable loop structure in the exposure of human immunodeficiency virus type 1 gp120 epitopes induced by receptor binding. J Virol 69: 5723–5733. 7543586 10.1128/jvi.69.9.5723-5733.1995 PMC189432

[13] Derdeyn CA, Decker JM, Bibollet-Ruche F, Mokili JL, Muldoon M, et al (2004) Envelope-constrained neutralization-sensitive HIV-1 after heterosexual transmission. Science 303: 2019–2022. 15044802 10.1126/science.1093137

[14] Wei X, Decker JM, Wang S, Hui H, Kappes JC, et al (2003) Antibody neutralization and escape by HIV-1. Nature 422: 307–312. 12646921 10.1038/nature01470

[15] Sagar M, Wu X, Lee S, Overbaugh J (2006) Human immunodeficiency virus type 1 V1-V2 envelope loop sequences expand and add glycosylation sites over the course of infection, and these modifications affect antibody neutralization sensitivity. J Virol 80: 9586–9598. 16973562 10.1128/JVI.00141-06 PMC1617272

[16] Liu Y, Curlin ME, Diem K, Zhao H, Ghosh AK, et al (2008) Env length and N-linked glycosylation following transmission of human immunodeficiency virus Type 1 subtype B viruses. Virology 374: 229–233. 18314154 10.1016/j.virol.2008.01.029 PMC2441482

[17] Bunnik EM, Pisas L, van Nuenen AC, Schuitemaker H (2008) Autologous neutralizing humoral immunity and evolution of the viral envelope in the course of subtype B human immunodeficiency virus type 1 infection. J Virol 82: 7932–7941. 18524815 10.1128/JVI.00757-08 PMC2519599

[18] Coetzer M, Cilliers T, Papathanasopoulos M, Ramjee G, Karim SA, et al (2007) Hum Longitudinal analysis of HIV type 1 subtype C envelope sequences from South Africa. AIDS Res Hum Retrov 23: 316–321. 10.1089/aid.2006.0207 17331039

[19] Gaschen B, Taylor J, Yusim K, Foley B, Gao F at al (2002) Diversity considerations in HIV-1 vaccine selection. Science 296: 2354–2360. 12089434 10.1126/science.1070441

[20] Zhang H, Hoffmann F, He J, He X, Kankasa C, et al (2005) Evolution of subtype C HIV-1 Env in a slowly progressing Zambian infant. Retrovirology 2: 67. 16274482 10.1186/1742-4690-2-67 PMC1308862

[21] Arien KK, Vanham G, Arts EJ (2007) Is HIV-1 evolving to a less virulent form in humans? Nat Rev Microbiol 5: 141–151. 17203103 10.1038/nrmicro1594 PMC7097722

[22] Frost SD, Wrin T, Smith DM, Kosakovsky Pond SL, Liu Y, et al (2005) Neutralizing antibody responses drive the evolution of human immunodeficiency virus type 1 envelope during recent HIV infection. Proc Natl Acad Sci USA 102: 18514–18519. 16339909 10.1073/pnas.0504658102 PMC1310509

[23] Patel MB, Hoffman NG, Swanstrom R (2008) Subtype specific conformational differences within the V3 region of subtype B and subtype C human immunodeficiency virus type 1 Env proteins. J Virol 82: 903–916. 18003735 10.1128/JVI.01444-07 PMC2224581

[24] Gnanakaran, S. Lang D, Daniels M, Bhattacharya T, Derdeyn CA, et al (2007) Clade-specific differences between human immunodeficiency virus type 1 clades B and C: diversity and correlations in C3-V4 regions of gp120. J Virol 81: 4886–4891. 17166900 10.1128/JVI.01954-06 PMC1900169

[25] Hioe CE, Wrin T, Seaman MS, Yu X, Wood B, et al (2010) Anti-V3 monoclonal antibodies display broad neutralizing activities against multiple HIV-1 subtypes. PLoS ONE 5: e10254. 20421997 10.1371/journal.pone.0010254 PMC2858080

[26] Felsovalyi K, Nadas A, Zolla-Pazner S, Cardozo T (2006) Distinct sequence patterns characterize the V3 region of HIV type 1 gp120 from subtypes A and C. AIDS Res Hum Retrov 22: 703–708. 10.1089/aid.2006.22.703 PMC1868395 16831095

[27] Rong R, Gnanakaran S, Decker JM, Bibollet-Ruche F, Taylor J, et al (2007) Unique mutational patterns in the envelope α2 amphipathic helix and acquisition of length in gp120 hypervariable domains are associated with resistance to autologous neutralization of subtype C Human Immunodeficiency Virus type 1. J Virol 81: 5658–5668. 17360739 10.1128/JVI.00257-07 PMC1900276

[28] Moore PL, Gray ES, Choge IA, Ranchobe N, Mlisana K, et al (2008) The C3-V4 region is a major target of autologous neutralizing antibodies in Human Immunodeficiency Virus type 1 subtype C infection. J Virol 82: 1860–1869. 18057243 10.1128/JVI.02187-07 PMC2258729

[29] Choisy M, Woelk CH, Guégan JF, Robertson DL (2004) Comparative study of adaptive molecular evolution in different Human Immunodeficiency Virus groups and subtypes. J Virol 78: 1962–1970. 14747561 10.1128/JVI.78.4.1962-1970.2004 PMC369455

[30] Yamaguchi-Kabata Y, Gojobori T (2000) Reevaluation of amino acid variability of the Human Immunodeficiency Virus type 1 gp120 envelope glycoprotein and prediction of new discontinuous epitopes. J Virol 74: 4335–4350. 10756049 10.1128/jvi.74.9.4335-4350.2000 PMC111951

[31] Olshevsky U, Helseth E, Furman C, Li J, Haseltine W, et al (1990) Identification of individual Human Immunodeficiency Virus type 1 gpl20 amino acids important for CD4 receptor binding. J Virol 64: 5701–5707. 2243375 10.1128/jvi.64.12.5701-5707.1990 PMC248709

[32] Rizzuto C, Sodroski J (2000) Fine definition of a conserved CCR5-binding region on the Human Immunodeficiency Virus type 1 glycoprotein gp120. AIDS Res Hum Retrov 16: 741–749. 10.1089/088922200308747 10826481

[33] Swaziland Demographic and Health Survey (2006–2007). Central Statistical Office, Ministry of Economic Planning and Development, Swaziland.

[34] Bernasconi D, Tavoschi L, Regine V, Raimondo M, Gama D, et al (2010) Identification of recent HIV infections and of factors associated with virus acquisition among pregnant women in 2004 and 2006 in Swaziland. J Clin Virol 48: 180–183. 20537582 10.1016/j.jcv.2010.04.010

[35] Suligoi B, Buttò S, Galli C, Bernasconi D, Salata RA, et al (2008) Detection of recent HIV infections in African individuals infected by HIV-1 non-B subtypes using HIV antibody avidity. J Clin Virol 41: 288–292. 18248848 10.1016/j.jcv.2007.11.020

[36] Hall TA (1999) Bioedit a user-friendly biological sequence alignment, editor and analysis program for Windows 95/98 NT. Nucl Acids Symp Ser 41: 95–98.

[37] Posada D, Crandall KA (1998) MODELTEST: testing the model of DNA substitution. Bioinformatics 14: 817–818. 9918953 10.1093/bioinformatics/14.9.817

[38] Jukes T, Cantor CR (1969) Evolution of protein molecules. In Mammalian protein metabolism. Edited by Munro HN. New York: Academic Press pp. 21–132.

[39] Bhatta H, Goldys EM (2009) Quantitative characterization of different strains of Saccharomyces yeast by analysis of fluorescence microscopy images of cell population. J Microbiol Methods 77: 77–84. 19318060 10.1016/j.mimet.2009.01.011

[40] Yang Z (1997) PAML: a program package for the phylogenetic analysis by maximum likelihood. Comput Appl Biosci 13: 555–556. 9367129 10.1093/bioinformatics/13.5.555

[41] Yang Z, Nielsen R, Goldman N, Pedersen AM (2000) Codon substitution models for heterogeneous selection pressure at amino acid sites. Genetics 155: 431–444. 10790415 10.1093/genetics/155.1.431 PMC1461088

[42] Anisimova M, Bielawsky JP, Yang Z (2001) Accuracy and power of likelihood ratio test in detecting adaptive evolution. Mol Biol Evol 18: 1585–1592. 11470850 10.1093/oxfordjournals.molbev.a003945

[43] Yang Z, Wong WS, Nielsen R (2005) Bayes empirical Bayes inference of amino acid sites under positive selection. Mol Biol Evol 22: 1107–1118. 15689528 10.1093/molbev/msi097

[44] Sokal RR (1981) Rohlf FJ. Biometry. W. H. Freeman and Company, New York, N.Y (1981)

[45] Huang CC, Tang M, Zhang MY, Majeed S, Montabana E, et al (2005) Structure of a V3-containing HIV-1 gp120 core. Science 310: 1025–1028. 16284180 10.1126/science.1118398 PMC2408531

[46] Auwerx J, François KO, Covens K, Van Laethem K, Balzarini J (2008) Glycan deletions in the HIV-1 gp120 V1/V2 domain compromise viral infectivity, sensitize the mutant virus strains to carbohydrate-binding agents and represent a specific target for therapeutic intervention. J Virol 382: 10–19. 10.1016/j.virol.2008.09.010 18930512

[47] Huang X, Jin W, Hu K, Luo S, Du T, et al (2012) Highly conserved HIV-1 gp120 glycans proximal to CD4-binding region affect viral infectivity and neutralizing antibody induction. Virology 423: 97–106. 22192629 10.1016/j.virol.2011.11.023

[48] Sharon RL, Kessler N, Levy R, Zolla-Pazner S, Görlach M, et al (2003) Alternative conformations of HIV-1 V3 loops mimic Β hairpins in chemokines, suggesting a mechanism for coreceptor selectivity. Structure 12: 225–236. 10.1016/s0969-2126(03)00011-x 12575942

[49] Tscherning C, Alaeus A, Fredriksson R, Björndal A, Deng H, et al (1998) Differences in chemokine coreceptor usage between genetic subtypes of HIV-1. Virology 241: 181–188. 9499793 10.1006/viro.1997.8980

[50] Cilliers T, Nhlapo J, Coetzer M, Orlovic D, Ketas T, et al (2003) The CCR5 and CXCR4 coreceptors are both used by human immunodeficiency virus type 1 primary isolates from subtype C. J Virol. 77: 4449–4456. 10.1128/JVI.77.7.4449-4456.2003 PMC150635 12634405

[51] Lin NH, Smeaton LM, Giguel F, Novitsky V, Moyo S, et al (2011) Prevalence and clinical associations of CXCR4-using HIV-1 among treatment-naive subtype C-infected women in Botswana. J Acquir Immune Defic Syndr 57: 46–50. 21346588 10.1097/QAI.0b013e318214fe27 PMC3353541

[52] Zhang M, Gaschen B, Blay W, Foley B, Haigwood N, et al (2004) Tracking global patterns of N-linked glycosylation site variation in highly variable viral glycoproteins: HIV, SIV, and HCV envelopes and Influenza hemagglutinin. Glycobiology 14: 1229–1246. 15175256 10.1093/glycob/cwh106

[53] Ogert RA, Lee MK, Ross W, Buckler-White A, Martin MA, et al (2001) N-linked glycosylation sites adjacent to and within the V1/V2 and the V3 loops of dualtropic human immunodeficiency virus type 1 isolate DH12 gp120 affect coreceptor usage and cellular tropism. J Virol 75: 5998–6006. 11390601 10.1128/JVI.75.13.5998-6006.2001 PMC114315

[54] Gray ES, Moore PL, Choge IA, Decker JM, Bibollet-Ruche F, et al (2007) Neutralizing antibody responses in acute human immunodeficiency virus type 1 subtype C infection. J Virol 81: 6187–6196. 17409164 10.1128/JVI.00239-07 PMC1900112

[55] Sterjovski J, Churchill MJ, Ellett A, Gray LR, Roche MJ, et al (2007) Asn 362 in gp120 contributes to enhanced fusogenicity by CCR5-restricted HIV-1 envelope glycoprotein variants from patients with AIDS. Retrovirology 4: 89. 18076768 10.1186/1742-4690-4-89 PMC2225424

[56] Peut V, Kent JS (2006) Fitness constraints on immune escape from HIV: Implications of envelope as a target for both HIV-specific T cells and antibody. Curr HIV Res 4: 191–197. 16611057 10.2174/157016206776055110

[57] Dacheaux L, Moreau A, Ataman-Onal Y, Biron F, Verrier B, et al (2004) Evolutionary dynamics of the glycan shield of the human immunodeficiency virus envelope during natural infection and implications for exposure of the 2G12 epitope. J Virol 78: 12625–12637. 15507649 10.1128/JVI.78.22.12625-12637.2004 PMC525068

[58] Carducci F, Marinozzi MC, Sampaolo M, Berrè S, Bagnarelli P, et al (2009) Dynamic features of the selection pressure on the Human Immunodeficiency Virus type 1 (HIV-1) gp120 CD4-binding site in a group of long term non progressor (LTNP) subjects. Retrovirology 6: 4. 19146663 10.1186/1742-4690-6-4 PMC2639529

[59] Aasa-Chapman MM, Seymour CR, Williams I, McKnight A (2006) Novel envelope determinants for CCR3 use by human immunodeficiency virus. J Virol 80: 10884–10889. 17041228 10.1128/JVI.01030-06 PMC1641764

[60] Kumar R, Tuen M, Li H, Tse DB, Hioe CE (2011) Improving immunogenicity of HIV-1 envelope gp120 by glycan removal and immune complex formation. Vaccine 29: 9064–9074. 21945958 10.1016/j.vaccine.2011.09.057 PMC3328143

[61] Li H, Chien PC Jr, Tuen M, Visciano ML, Cohen S, et al (2008) Identification of an N-linked glycosylation in the C4 region of HIV-1 envelope gp120 that is critical for recognition of neighboring CD4 T cell epitopes. J Immunol 180: 4011–4021. 18322210 10.4049/jimmunol.180.6.4011

[62] Korber BTM, MacInnes K, Smith RF, Myers G (1994) Mutational trends in V3 loop protein sequences observed in different genetic lineages of Human Immunodeficiency Virus type 1. J Virol 68: 6730–6744. 8084005 10.1128/jvi.68.10.6730-6744.1994 PMC237094

[63] Kumar R, Tuen M, Li H, Tse DB, Hioe CE (2011) Improving immunogenicity of HIV-1 envelope gp120 by glycan removal and immune complex formation. Vaccine 29: 9064–9074. 21945958 10.1016/j.vaccine.2011.09.057 PMC3328143

